# Zn^2+^ dependent glyoxalase I plays the major role in methylglyoxal detoxification and salinity stress tolerance in plants

**DOI:** 10.1371/journal.pone.0233493

**Published:** 2020-05-26

**Authors:** Rituraj Batth, Muskan Jain, Ashish Kumar, Preeti Nagar, Sumita Kumari, Ananda Mustafiz

**Affiliations:** 1 Plant Molecular Biology Laboratory, Faculty of Life Sciences and Biotechnology, South Asian University, Chanakyapuri, New Delhi, India; 2 School of Biotechnology, Sher-e-Kashmir University of Agricultural Sciences and Technology, Jammu, JK, India; University of Delhi, INDIA

## Abstract

Glyoxalase pathway is the major pathway of methylglyoxal detoxification and is ubiquitously present in all organisms ranging from prokaryotes to eukaryotes. Glyoxalase I (GLYI) and Glyoxalase II (GLYII), the two core enzymes of this pathway work together to neutralize methylglyoxal (MG), a dicarbonyl molecule with detrimental cytotoxicity at higher concentrations. The first step towards the detoxification of MG is catalyzed by GLYI, a metalloenzyme that requires divalent metal ions (either Zn^2+^ as seen in eukaryotes or Ni^2+^ as in prokaryotes). However, both Zn^2+^ and Ni^2+^ dependent GLYIs have been shown to co-exist in a higher eukaryote i.e. *Arabidopsis thaliana*. In the present study, we determine the role of both Zn^2+^ dependent (AtGLYI2) and Ni^2+^ dependent (AtGLYI3, AtGLYI6) GLYIs from Arabidopsis in salinity stress tolerance. AtGLYI2 overexpressing Arabidopsis plants showed better growth rate while maintaining lower levels of MG under high saline conditions. They were taller with more number of silique formation with respect to their Ni^2+^ dependent counterparts. Further, lack in germination of Arabidopsis AtGLYI2 mutants in presence of exogenous MG indicates the direct involvement of Zn^2+^ dependent GLYI in MG detoxification, suggesting Zn^2+^ dependent GLYI as the main enzyme responsible for MG detoxification and salinity stress tolerance.

## Introduction

Methylglyoxal (MG) is a dicarbonyl molecule produced as a by-product of various metabolic pathways such as glycolysis, lipid peroxidation and amino acid metabolism. Levels of MG have been found to increase by 2–6 folds in response to abiotic stresses in plant system [1]. At higher concentration, it acts as a potent cytotoxic molecule which reacts with major macromolecules to forms advanced glycation end products [2] and causes inactivation of proteins and leads to oxidative damage of cellular components [3].

Glyoxalase system plays a major role in detoxification of MG. It consists of two main enzymes, glyoxalase I (GLYI) and glyoxalase II (GLYII). GLYI, using reduced glutathione (GSH) as cofactor converts MG to S-D-lactoylglutathione (SLG), GLYII then acts upon SLG and hydrolyzes it to D-lactate with the release of one molecule of GSH. Since substrate for GLYI is a complex formed between GSH and MG, detoxification of MG is strongly dependent on the availability of cellular GSH. A deficiency in GSH strongly limits the formation of hemithioacetal, leading to MG accumulation and in turn causing cellular damage. Apart from glyoxalase several other enzymes also work to detoxify MG, for example, an enzyme named glyoxalase III (GLYIII) converts MG directly into D-lactate in a single step without using GSH as a cofactor [4].

The glyoxalase system has been extensively studied due to its potential role in stress management in all living organisms ranging from bacteria to humans [5,6]. Glyoxalase pathway in plants has been associated with various abiotic stresses. The expression of GLYI and GLYII has been found to up-regulate under multiple abiotic stress in rice and other plant species [7] and both MG and glyoxalases have been suggested as a biomarker for stress tolerance [8].

Glyoxalase enzymes have been extensively characterized in various species. GLYI is broadly classified into two metal activation classes, i.e., Zn^2+^ dependent and Ni^2+^ dependent [9]. All Ni^2+^ dependent GLYI were thought to be of prokaryotic origin, and all Zn^2+^ dependent GLYIs were thought to be of eukaryotic origin. However, recent studies refuted this notion. Multiple GLYI encoding genes from both metal activation classes are present in *P*. *aeruginosa* [10]. A unique MG inducible Ni^2+^ dependent GLYI was reported in rice [11]. A study reported the co-existence of both metal-dependent GLYIs in a higher eukaryote, i.e., *Arabidopsis thaliana* [12]. Out of three active GLYIs in Arabidopsis, AtGLYI2 is Zn^2+^ dependent whereas AtGLYI3 and AtGLYI6 are Ni^2+^ dependent enzymes. The subcellular localization study showed that AtGLYI2 consist of 28 amino acid long nuclear localization signal (NLS) at N-terminus and similar to rice GLYI (OsGLYI-8), it is localized in the nucleus [13]. The kinetic profile of these enzymes revealed AtGLYI2 to be the most active with 250 and 670 times more activity than AtGLYI3 and AtGLYI6 [12]. Also, so far AtGLYI2 is reported to have the highest specific activity than GLYI from any other plant species. Among Ni^2+^ dependent GLYIs, AtGLYI3 and AtGLYI6 were found to be most active in comparison to GLYIs from rice, *P*. *aeruginosa*, *E*. *coli* and *C*. *acetobutylicum* [12]. The role of these AtGLYI genes in multiple abiotic stress tolerance was evaluated in *E*. *coli* and yeast. It was found that AtGLYI2 has more important role in MG detoxification as well as in providing tolerance against multiple abiotic stresses in comparison to AtGLYI3 and AtGLYI6 genes [12]. This study suggests that Zn^2+^ dependent AtGLYI contributes more towards stress tolerance than Ni^2+^ dependent AtGLYI. These results further led us to validate the role of these GLYI genes (AtGLYI2, AtGLYI3, AtGLYI6) in plant system.

In the present study, the mutant and overexpressing Arabidopsis plants of AtGLYI2, AtGLYI3 and AtGLYI6 have been studied. The in-depth study reveals that Arabidopsis transgenic plants overexpressing AtGLYI2, AtGLYI3 and AtGLYI6 genes provide tolerance against salinity stress and out of the three, AtGLYI2 overexpressing plants are the tallest and produce the maximum number of siliques under both stress and control conditions. Apart from this, we also report that AtGLYI2 could be the main enzyme directly involved in the detoxification of MG as well as in conferring tolerance to salinity stress.

## Materials and methods

### Growth of plant material

The plant lines used in this study include *Arabidopsis thaliana* (Col-0 ecotype), Transgenic line for AtGLYI2 (T-GLYI2), AtGLYI3 (T-GLYI3) and AtGLYI6 (T-GLYI6) and T-DNA insertion lines M-GLYI2-2 (SALK_131547), M-GLYI3-2 (SALK_110070C) and M-GLYI6-2 (SALK_010206). All the T-DNA insertion lines for *AtGLYI2*, *AtGLYI3* and *AtGLYI6* were ordered from The Arabidopsis information resource (TAIR, https://www.arabidopsis.org/). They were PCR screened using genome specific and left border primer. Homozygous plants for all three *AtGLYI* genes were identified and used in the study. All plants were grown in growth chamber at 22°C with photoperiod of 16 h and humidity of 60% and day cycle of 16 h and night cycle of 8 h. The seeds were sterilized in 70% ethanol followed by treatment with mixture of 2.5% bleach+ 0.1% Triton-X. Sterilized seeds were then vernalized for 3 days at 4°C. Autoclaved soil mixture in 4:3:2 ratios of soilrite: vermiculite: agropeat was used to grow Arabidopsis plants.

### Cloning of *AtGLYI2*, *AtGLYI3* and *AtGLYI6* genes into destination vector

Total RNA was isolated from fresh Arabidopsis leaf tissue using IRIS Kit (Bangalore, Genei) according to manufacturer’s instructions (S1 Fig). The RNA was reverse transcribed using RevertAid H Minus first stand cDNA synthesis Kit (Fermentas Life Sciences, USA). The first strand of cDNA was used to amplify *AtGLYI2* (AT1G08110.1), *AtGLYI3* (AT1G11840.1) and *AtGLYI6* (AT1G67280.1) genes with Q5 polymerase (NEB) using gene specific forward and reverse primer containing attB1 and attB2 sequences for subsequent cloning into donor vector (S1 Table & S1 Fig). Full-length *AtGLYI* genes were recombined into pDONOR221 vector (Addgene) using BP clonase II enzyme (Invitrogen, instructions for cloning followed as per manufacturer’s protocol). Sequence of all the three *AtGLYI* genes recombined into pDONOR vector were confirmed by Sanger sequencing, only the correct sequences were further recombined into pEARLEY100 [14], a plant expression vector in presence of LR clonase II enzyme (Invitrogen, instructions for cloning followed as per manufacture’s protocol).

### Generation of Arabidopsis transgenic plants overexpressing *AtGLYI2*, *AtGLYI3* and *AtGLYI6* genes

The recombinant plasmids pEARLEY100+*AtGLYI2*, pEARLEY100+*AtGLYI3* and pEARLEY100+*AtGLYI6* were transformed into *Agrobacterium tumefaciens* (GV3101) and selected on LB plates containing Rifampicin (10 μg/ml), Gentamycin (50 μg/ml) and Kanamycin (50 μg/ml). Model plant *Arabidopsis thaliana* (Col-0 ecotype) at 20–30% flowering stage were individually transformed with recombinant plasmids via Agrobacterium mediated floral dip method [15]. The plants were watered with MS medium and grown normally for 1 month. Watering was withheld when siliques started to turn brown, followed by seed harvesting.

### Confirmation of transgenic plants at genomic DNA and protein level

For screening of homozygous single insertion transgenic lines, the genomic DNA was isolated from the leaf tissue (300 mg) of wild type and the three respective transgenic plants using CTAB method. All genomic DNA samples were PCR-screened using 35S CaMV promoter specific forward primer (FP) and *AtGLYI2*, *AtGLYI3* and *AtGLYI6* gene specific reverse primers (RP). Primers used are listed in S2 Table.

For protein isolation, 300 mg of leaf tissue was processed according to the protocol [16]. The protein was then quantified using Bradford assay. 20 μg of the total protein was separated by SDS-PAGE and transferred onto PVDF membrane. Polyclonal antibody [12] raised in rabbit against purified AtGLYI2, AtGLYI3 and AtGLYI6 protein was used to detect specific proteins. The protein bands of 27kDa, 35kDa and 43kDa were visualized for AtGLYI2, AtGLYI3 and AtGLYI6 proteins, respectively by Pierce ECL western blotting substrate as per manufacturer’s protocol.

### Effects of exogenous methylglyoxal on the growth of Arabidopsis transgenic and mutant plants

To study the effect of methylglyoxal (MG), WT, transgenic (T-GLYI2, T-GLYI3 and T-GLYI6) and mutant (M-GLYI2, M-GLYI3 and M-GLYI6) seeds were germinated on ½ MS agar with 2.5% sucrose and 0.05% of plant preservative mixture (PPM), supplemented with MG (0.25 mM, 0.5 mM and 1.0 mM) for 2 weeks. The plants grown on plain ½ MS medium were used as experimental controls.

### Testing Arabidopsis transgenic plants for their tolerance towards salinity stress

To study the tolerance level, 1 month old transgenic (T-GLYI2, T-GLYI3 and T-GLYI6) and WT plants were irrigated with water (control) or 150 mM and 300 mM NaCl (salinity stress) for a period of 10 days. Growth parameters like silique number and plant height were measured for tolerance assessment.

### Measurement of methylglyoxal (MG) levels

Methylglyoxal content was measured by the described protocol in Jain et al., [17]. Sample of leaf tissue (300 mg) was taken in mortar and homogenized to fine powder in liquid nitrogen. 2 ml of 0.5 M perchloric acid was added, mixed and allowed to thaw. The extract was kept on ice for 15 min and centrifuged at 13000 rpm for 10 min at 4°C. The supernatant was transferred in fresh tube and neutralized by the gradual addition of 1 M Na_2_HPO_4_ and pH was checked on pH strip to confirm its neutralization and incubated at room temperature for 15 min followed by centrifugation at 13000 rpm for 10 min at 4°C. The reaction mixture (1 ml) contained 250 μl of 7. 2 mM 1,2-dia-aminobenzene, 100 μl of 5 M perchloric acid, 10 μl of 100 mM NaN_3_ (to inhibit peroxidase), and 650 μl of neutralized supernatant. Absorbance of the reaction mixture was initially taken at 0 h, 336 nm and used as blank. The same mixture was incubated at room temperature for 3 h and absorbance was again taken at 336 nm. MG content was calculated by using standard curve and expressed as μmol gm^-1^ leaf fresh weight.

## Results

### Confirmation of AtGLYI2, AtGLYI3 and AtGLYI6 overexpressing transgenic Arabidopsis plants at genomic DNA and protein level

**Fig 1:**
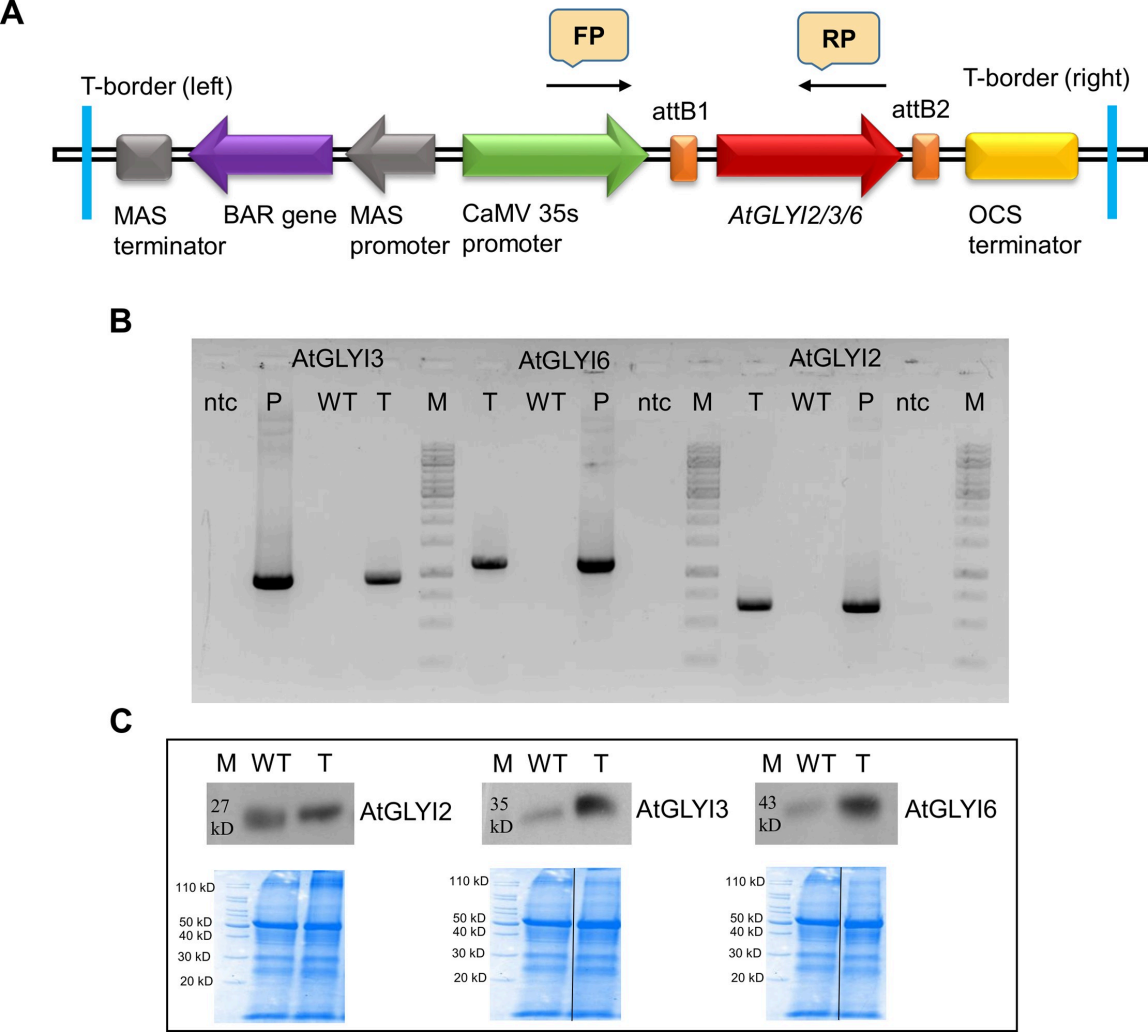
Confirmation of *AtGLYI2*, *AtGLYI3* and *AtGLYI6* transgenic *Arabidopsis* plants. (A) Schematic representation of pEARLEY100+AtGLYI2/3/6 (expression clone) construct individually used for transformation of *Arabidopsis thaliana* plant via floral-dip (B) Putative single insertion homozygous plants (T4 generation transgenic plants) were screened by polymerase chain reaction (PCR) using 35s CaMV promoter specific forward primer (FP) and *AtGLYI2*, *AtGLYI3* and *AtGLYI6* gene specific reverse primers (RP). (C) Western blot was performed to check the expression of *AtGLYI2*, *AtGLYI3* and *AtGLYI6* in wild type (WT) and transgenic plants. The control SDS coommassie blue gel lanes have been rearranged from the original gel (S1 Raw Images) and separated by black line for representation purpose. Here, WT: wild-type, T-transgenic, P: positive control; ntc: non-template control.

*AtGLYI2*, *AtGLYI3* and *AtGLYI6* gene overexpressing single insertion homozygous transgenic lines were screened to confirm the insertion of T-DNA in the genome of *Arabidopsis thaliana* plant (Fig 1A). The amplified PCR products showed the presence of approximately 558 bp, 853 bp and 1053 bp bands for AtGLYI2, AtGLYI3 and AtGLYI6 genes from respective transgenic plants and no amplified DNA fragment from wild-type plants confirmed the insertion of T-DNA fragment into the genome of *Arabidopsis thaliana* (Fig 1B).

Further, overexpression of AtGLYI2, AtGLYI3 and AtGLYI6 proteins in transgenic plants with respect to WT Arabidopsis was also checked by western blotting. Anti-rabbit polyclonal primary antibody [12] designed against AtGLYI2, AtGLYI3 and AtGLYI6 protein was used to detect the expression of AtGLYI proteins in WT and transgenic plants. For experimental control, we used coomassie blue stained SDS PAGE gel showing protein profile of wild-type and transgenic plants (Fig 1C). Expected bands of 27 kD, 35 kD and 42 kD for AtGLYI2, AtGLYI3, and AtGLYI6 proteins were detected in both transgenic and wild-type plants. AtGLYI2 has single domain and size of 27 KD while AtGLYI3 and AtGLYI6 have two domains and size of 37 and 42 KD, respectively [12] (S2 Fig). However, the presence of darker intensity bands in transgenic plants confirmed the higher expression of AtGLYI2, AtGLYI3 and AtGLYI6 proteins in transgenic plants than wild-type plants (Fig 1C).

### Confirmation of AtGLYI2, AtGLYI3 and AtGLYI6 mutant Arabidopsis plants at genomic DNA level

**Fig 2:**
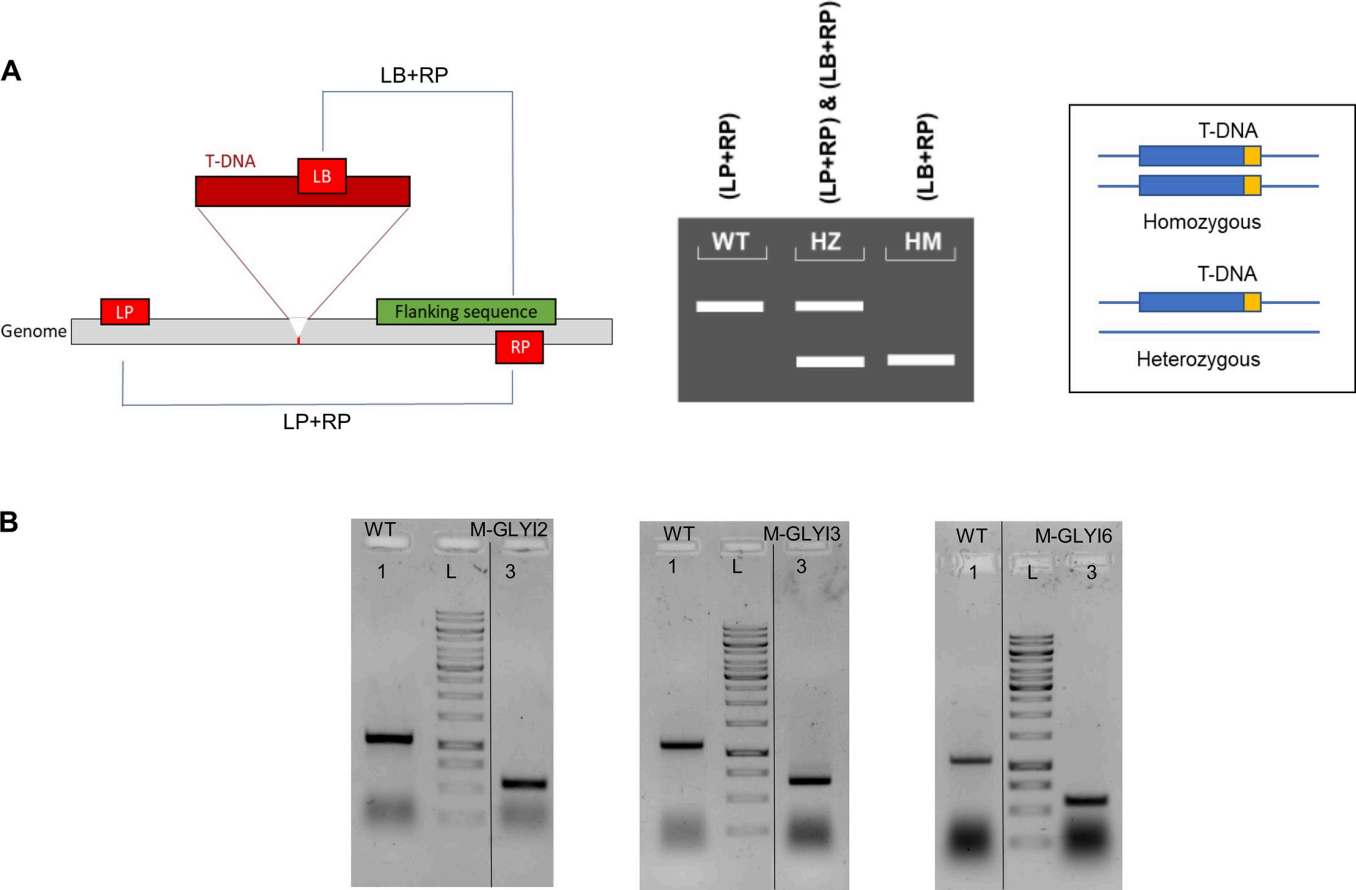
PCR genotyping for analysis of T-DNA insertion mutant plants at DNA level. (A) Schematic diagram showing primer combinations for screening T-DNA insertion mutants. Amplification product from a heterozygous individual should give bands from both LP+RP and LB+RP primer combinations as it contains single T-DNA insert in either of the alleles. Amplification from a homozygous individual is expected only from LB+RP primer combination as it has T-DNA inserted in both copies of the gene, and WT plants would give a higher size band with LP+RP. Here, LP: Left primer; RP: Right primer and LB: Left border primer, HZ: Heterozygous, HM: Homozygous. (B) Using genomic DNA as template, a single PCR reaction with LP+RP+LB primers was set for each of the three mutant plants M-GLYI2, M-GLYI3 & M-GLYI6 obtained from ABRC along with WT. Amplified products were loaded in the same lane of 1% agarose gel to confirm the genotype of mutant plants. The lanes of the gel are the representation of original gel image (S1 Raw Images) rearranged for figure preparation and the separation between them has been marked by a vertical black line. All mutants used in this study were homozygous for the mutation. Here L: 1 kb ladder (GeneRuler).

For confirming the genotype of the mutant plants, 2 step PCR genotyping tool was used, where first PCR primers were designed using SALK T-DNA primer design tool http://signal.salk.edu/tdnaprimers.2.html (S3 Table). Three sets of primers were used for this experiment i.e. left primer (LP), right primer (RP) that spans the predicted T-DNA insertion site and border primer (LB) designed from the left-border of T-DNA. In the first PCR reaction, gene specific primer (LP+RP) were used to detect the presence of wild-type copy in the plant. Amplification product of approx. 900 bp was obtained from a heterozygous individual or wild-type plant (Fig 2A). No band was amplified for homozygous plant, as both copies of the gene were replaced by T-DNA inserts (Fig 2A). A second PCR reaction with LB+RP primer gives amplification in both homozygous and heterozygous individuals with amplified product size being approx. equal to >500 bp (Fig 2A). The presence of bands corresponding to specific size after PCR for AtGLYI2, AtGLYI3 and AtGLYI6 mutants confirmed the mutants as homozygous (Fig 2B).

### AtGLYI2 transgenic plants grow better than AtGLYI3 and AtGLYI6 transgenics in presence of methylglyoxal

**Fig 3:**
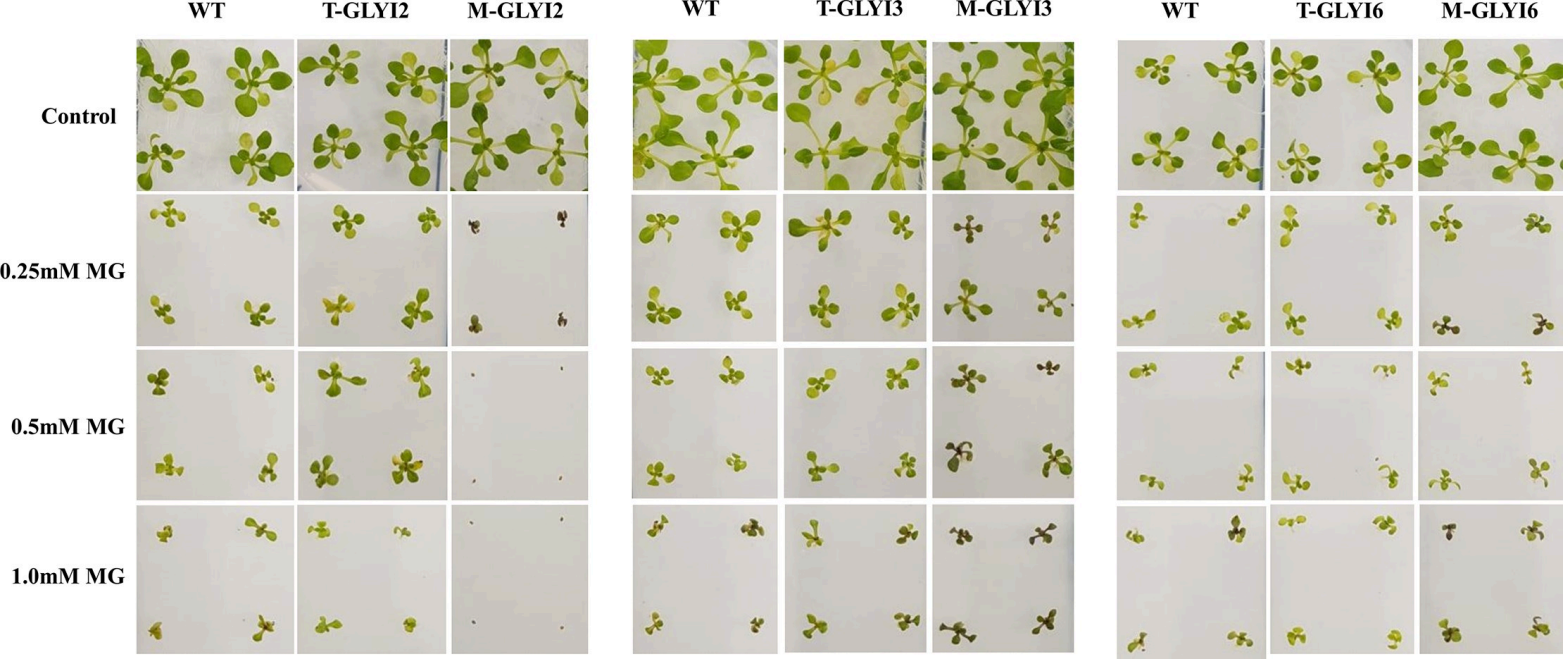
Effects of MG on plant growth. Two-week-old wildtype, transgenic (T-GLYI2/3/6) and mutant (M-GLYI2,3,6) Arabidopsis plants grown in ½ MS media supplemented with 2.5% sucrose and 0.25 mM, 0.5 mM and 1.0 mM concentrations of MG.

Seeds from all plants were germinated in ½ MS medium supplemented with different concentrations of MG and grown for 2 weeks. Under standard growth conditions AtGLYI2, AtGLYI3 and AtGLYI6 transgenic plants and mutant seedlings showed no striking difference regarding development and morphology when compared to the wild-type plants (Fig 3), however mutant plants with deleted *AtGLYI6* gene seem to grow slightly better under control conditions. However, in the presence of MG, the growth of all plants was compromised.

Out of all the three mutant plants, the growth of AtGLYI2 mutant plants was severely affected in the presence of MG. AtGLYI2 mutant seedlings germinated and survived only till four-leaf stage at 0.25 mM concentration of MG whereas at higher concentrations (0.5 and 1.0 mM) of MG AtGLYI2 mutants failed to germinate (Fig 3). Unlike AtGLYI2, the seedling establishment of both AtGLYI3 and AtGLYI6 mutant plants was not affected, instead they showed germination in the presence of 0.5 mM and 1.0 mM of MG (Fig 3). On the contrary, WT Arabidopsis plants could grow better under the same conditions. This highlights the point that knock-out/ knock-down of either of the AtGLYI genes has drastic effects on germination and seedling establishment in presence of cytotoxic methylglyoxal (MG).

However, seedling establishment of AtGLYI2 mutant was most severely affected in presence MG, which was not the case with AtGLYI3 and AtGLYI6 mutant plants. These results indicate that elimination of MG is mostly dependent on AtGLYI2 enzyme and it could play a major role in its detoxification *in vivo*.

### Transgenic plants over-expressing AtGLYI2 gene show better tolerance towards salinity stress

Stress tolerance potential of AtGLYI2, AtGLYI3, and AtGLYI6 transgenic plants were tested under saline conditions. 30 days old soil-grown plants were irrigated with 150 mM and 300 mM NaCl solution for 10 days, whereas plants irrigated with normal tap water were used as experimental control.

**Fig 4:**
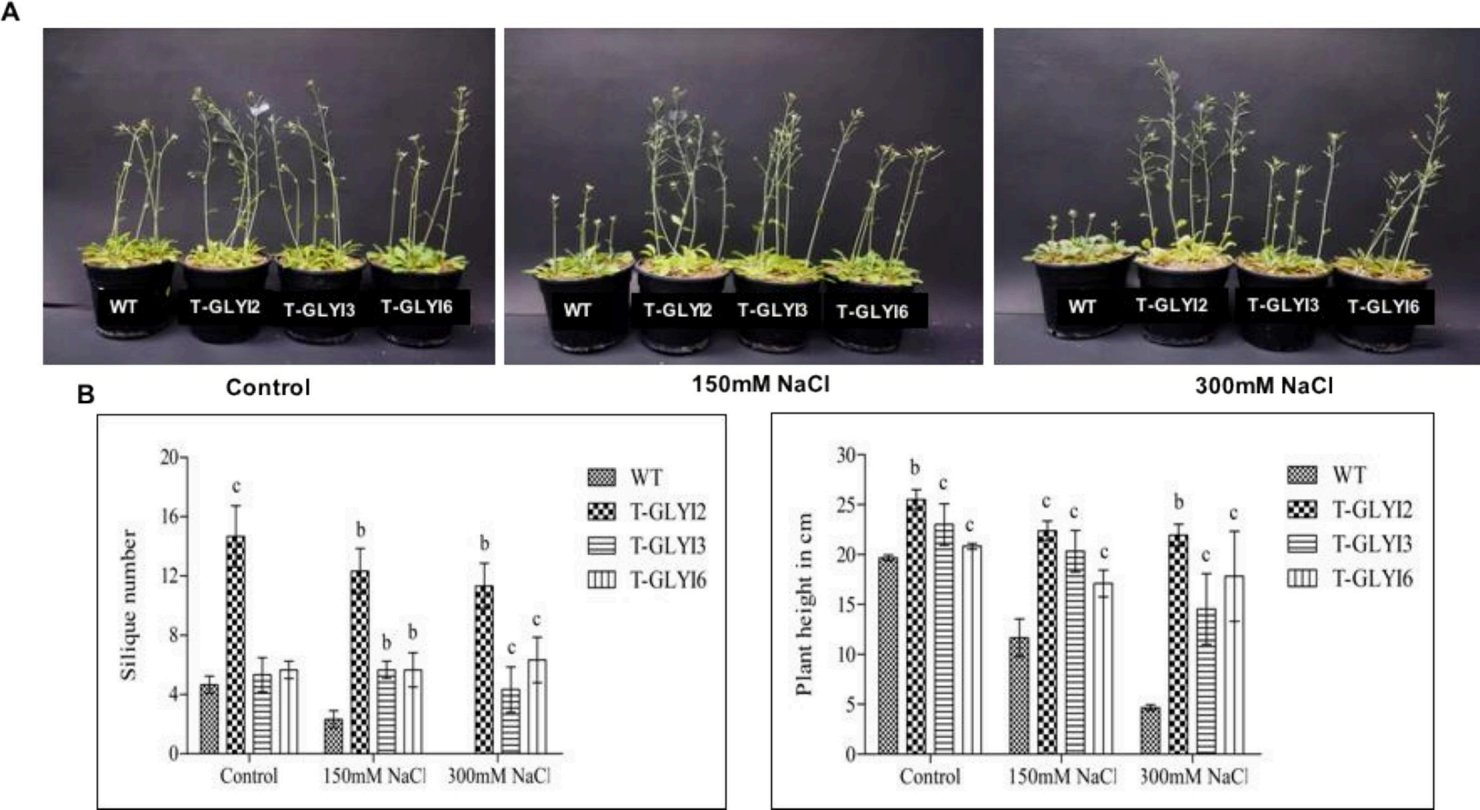
Phenotypic analysis of *AtGLYI2*, *AtGLYI3* and *AtGLYI6* transgenic lines under salt stress. (A) Thirty days old soil grown *Arabidopsis* plants were treated with 150mM and 300mM NaCl for a period of 10 days. (B) Growth and yield parameters such as plant height and silique number were measured from two independent transgenic lines and their cumulative readings are represented as mean ± standard deviation (*STD*; *n = 3*). Statistically significant difference was determined using two-tailed Student’s t-test as compared with wild-type plants under similar conditions shown as ^*a*^*P<0*.*001*, ^*b*^*P<0*.*01*, ^*c*^*P<0*.*05*. Here, WT: wild type; T-GLYI2, T-GLYI3 and T-GLYI6 stands for T4 generation transgenic plants ectopically expressing *AtGLYI2*, *AtGLYI3* and *AtGLYI6* genes, respectively.

It was observed that under control conditions, no significant difference regarding morphology was seen between WT and transgenic plants. However, plants overexpressing either AtGLYI2, AtGLYI3 or AtGLYI6 genes could grow much better than wild-type under both 150 mM and 300 mM NaCl stress (Fig 4A and 4B). The growth of wild-type plants was stunted and severely affected regarding plant height and yield, whereas the transgenic plants grew normally and produced siliques even under salinity stress.

Among all the three transgenic lines, AtGLYI2 overexpressing plants were the tallest and produced maximum number of siliques under both saline and control conditions. The number of siliques in WT plants showed around 46% reduction in response to 150 mM of NaCl and produced no siliques at all when grown in the presence of 300 mM of NaCl, while AtGLYI2 transgenic plants showed slight reduction in silique number under similar conditions as compared with the respective control counterparts.

### AtGLYI transgenic lines maintain lower MG levels under salinity stress

**Fig 5:**
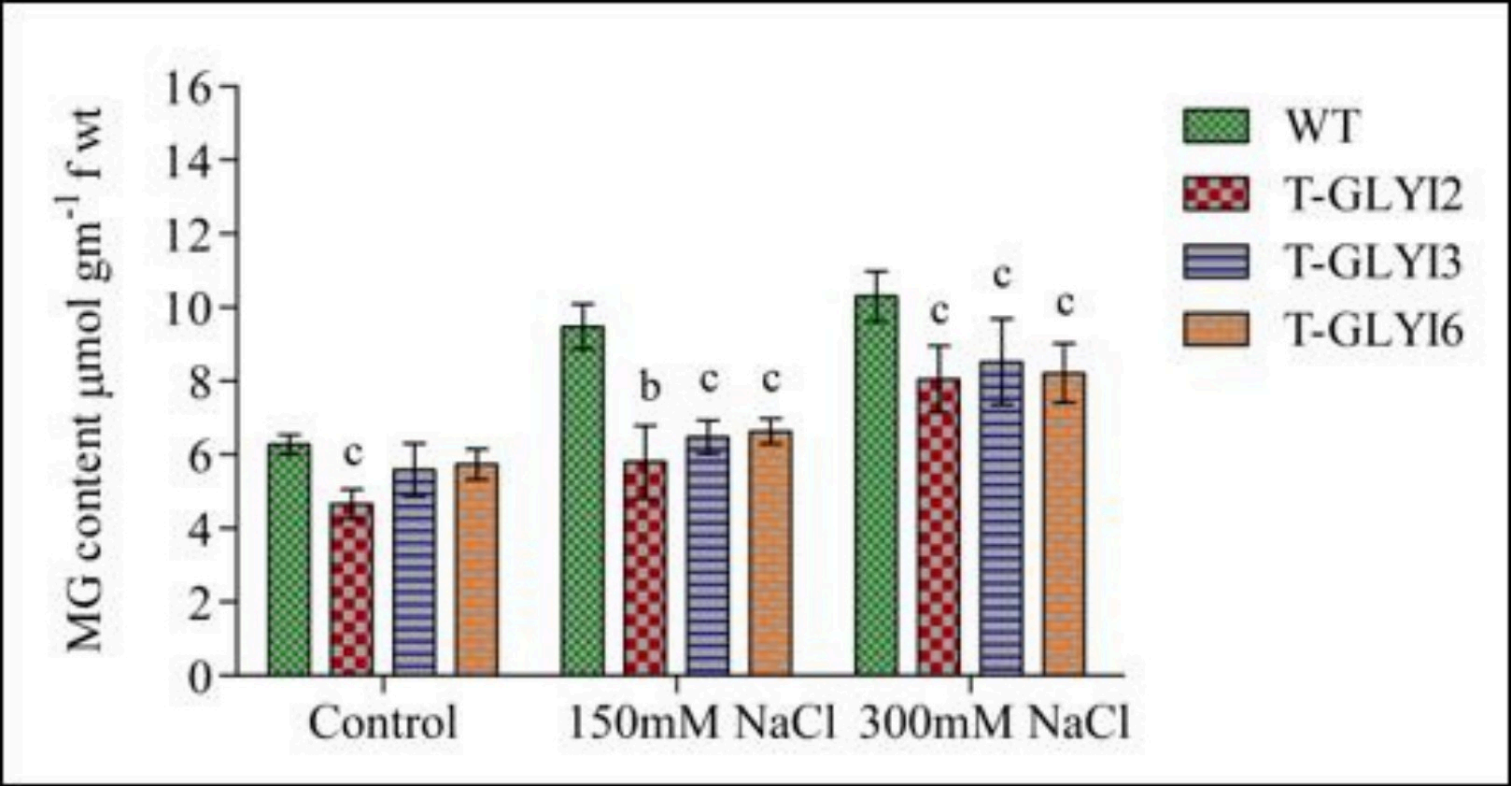
Determination of levels of MG in control and stress treated *Arabidopsis* plants. Comparing level of MG in wild-type and *AtGLYI2*, *AtGLYI3* and *AtGLYI6* overexpressing transgenic plants grown under control as well as 150mM and 300mM of NaCl stress conditions. MG content from two independent transgenic lines was measured and their combined readings are represented as mean ± standard deviation (*STD*; *n = 3*). Statistically significant difference was determined using two-tailed paired Student’s t-test as compared to WT plants under same conditions and indicated by ^*a*^*P<0*.*001*, ^*b*^*P<0*.*01*, ^*c*^*P<0*.*05*. Here, WT: wild-type, T-GLYI2, T-GLYI3 and T-GLYI6 stands for T4 generation transgenic plants ectopically expressing *AtGLYI2*, *AtGLYI3* and *AtGLYI6* genes, respectively.

As the glyoxalase pathway has a direct correlation with concentrations of MG in the cells, its level was measured in WT and AtGLYI transgenic plants in response to salinity stress (Fig 5). WT plants showed a sharp increase in MG content (37–61%), whereas AtGLYI2, AtGLYI3, and AtGLYI6 transgenic plants showed around 9–37% increase in MG content when exposed to 150 mM and 300 mM of NaCl for a period of 10 days. It is also worth noting that level of MG in AtGLYI2 overexpressing transgenic plants was always maintained slightly lower than AtGLYI3 and AtGLYI6 overexpressing transgenic plants under both control and stress conditions.

## Discussion

Glyoxalase pathway, mainly GLYI and GLYII, has been well correlated with abiotic stress [18,19,20,7,11]. It has also been established that in response to salinity and other abiotic stresses methylglyoxal level increases [1]. Glyoxalase I (GLYI) is the first enzyme of the glyoxalase pathway and catalyzes the isomerization of non-enzymatically formed hemithioacetal (HTA) of MG and GSH to S-D lactoylglutathione (SLG) [21,22,23,24,9]. It is metalloenzymatic and requires divalent metal ions for its activity and is broadly classified into two metal activation classes, i.e., Zn^2+^ dependent and non-Zn^2+^ dependent [9].

Previous studies have shown that overexpression of GLYI imparts tolerance against various abiotic stress in plants. For example, overexpressing glyoxalase I gene from *Brassica juncea*, *Vigna mungo*, *Arabidopsis thaliana* and *Oryza sativa* provides significant tolerance towards salinity stress [18,25,26,11] Also, tolerance to salt, heavy metal and drought stress was observed upon overexpressing GLYI in *B*. *juncea* [27]. These and various other studies indicate the significant role of glyoxalase pathway in stress, but none shed light on its correlation with the metal dependency of GLYI. However, a study conducted in tobacco showed the role of unique Ni^2+^ dependent rice GLYI in providing tolerance against salt, oxidative, osmotic and exogenous MG [11]. This was the first study to relate the role of metal dependent GLYI (a possible relic of prokaryotic origin) with abiotic stress.

Higher eukaryotes such as plants consists of multiple isoforms of GLYI proteins [7,28,29]. Interestingly most of the plant species have both Ni^2+^ and Zn^2+^ dependent GLYI [30]. But it is still unclear which particular GLYI might play the major role in MG detoxification and abiotic stress tolerance. Based on the genome wide study carried out in *Arabidopsis thaliana*, eleven members of GLYI gene family were identified. All these GLYI gene family members were differentially regulated in response to stress conditions and various developmental stages [7]. Further *in silico* analysis revealed that out of eleven, only three GLYI genes were active and contained all domains required for glyoxalase activity [12]. These three active GLYIs are: AtGLYI2, AtGLYI3, and AtGLYI6. Out of these three genes, AtGLYI2 is Zn^2+^ dependent and AtGLYI3 and AtGLYI6 are Ni^2+^ dependent. The association of these metal-dependent AtGLYI genes with stress tolerance has previously been evaluated in *E*. *coli* where it was shown that AtGLYI2 overexpression in *E*. *coli* provided better tolerance against salinity, oxidative, methylglyoxal, osmotic and heat stress in comparison to AtGLYI3 and AtGLYI6 genes [12]. These results further led us to validate the role of both Zn^2+^ and Ni^2+^ dependent AtGLYI genes in a model plant system, *Arabidopsis thaliana*. In this study the model system Arabidopsis was chosen to decipher the individual role of different GLYI in MG detoxification and salinity stress tolerance.

Exogenous application of MG has detrimental effect on the growth of plants [11]. We therefore, analyzed the comparative effect of exogenous MG on growth of AtGLYI2, AtGLYI3 and AtGLYI6 overexpressing single insertion homozygous transgenic and mutant plants. The growth of AtGLYI2 and AtGLYI3 overexpressing transgenic plants was better than wild-type and mutant plants. Both AtGLYI2 and AtGLYI3 transgenic plants grew better and were bigger in size with slight or no discoloration of leaves as compared to WT plants in the presence of 0.25 mM and 0.5 mM of MG (Fig 3A and 3B). GLYI mutant plants were differentially sensitive to exogenous MG application. AtGLYI2 mutant plants were highly sensitive to the presence of MG during both germination and post-germinative development. The growth of AtGLYI2 mutant plants was severely affected in the presence of MG. The seedlings of AtGLYI2 mutants germinated and survived only till four-leaf stage at 0.25 mM concentration of MG whereas, they failed to germinate at higher concentrations (0.5 and 1.0 mM) of MG (Fig 3A). Unlike AtGLYI2, the seedling establishment of both AtGLYI3 and AtGLYI6 mutant plants was not affected, rather they showed germination in the presence of 0.5 mM and 1.0 mM of MG (Fig 3B and 3C). The high sensitivity of AtGLYI2 mutant during seedling establishment when grown in the presence of MG indicates towards the direct involvement of AtGLYI2 in MG detoxification. Differential germination and growth of these plants suggests that out of the three Arabidopsis GLYI, AtGLYI2 is the main enzyme involved in the detoxification of MG. The reason for AtGLYI3 and AtGLYI6 mutant lines to be less sensitive to MG could be due to an overlapping high enzymatic activity of AtGLYI2 enzyme, as the kinetic profile reveals AtGLYI2 to be 250 and 670 times more active than AtGLYI3 and AtGLYI6, respectively [12]. Additionally, AtGLYI3 and AtGLYI6 may possibly be primarily involved in detoxification of carbonyl species other than MG during germination and seedling establishment stage. A recent study has shown that AtGLYI3 (GLXI:1) and AtGLYI6 (GLXI:2) possess the specific feature of switching substrate specificity depending on metal ion cofactor being used. They prefer Ni^2+^ in the conversion of MG-GSH and Mn^2+^ ion in the conversion of (glyoxal) GO-GSH [31].

Arabidopsis plants overexpressing AtGLYI2 (Zn^2+^ dependent), AtGLYI3 and AtGLYI6 (Ni^2+^ dependent) genes could withstand high saline conditions. Among all the three transgenic lines, AtGLYI2 overexpressing plants were the tallest and produced maximum number of siliques under both saline (150 mM and 300 mM NaCl) and control conditions. The number of siliques in WT plants showed 46% reduction in response to 150 mM of NaCl and produced no siliques at all when grown in presence of 300 mM of NaCl, while AtGLYI2 transgenic plants showed slight reduction in silique number under similar conditions as compared with the respective control counterparts (Fig 4). AtGLYI3 and AtGLYI6 transgenic plants were able to maintain the silique number even under 300 mM salinity stress however the number was less as compared to AtGLYI2.

The glyoxalase pathway has a direct correlation with the concentration of MG in the cell. Therefore, MG level was measured in both WT and transgenic Arabidopsis plants under control conditions and upon exposure to salt stress. All three transgenic plants maintained lower levels of MG, whereas excess accumulation of MG was observed in WT plants (Fig 5). MG accumulation was not completely abolished in stressed AtGLYI transgenic plants and a basal level of MG was maintained similar to control conditions. This emphasizes that MG might play a role in signaling. MG has been shown to affect global gene expression in rice which indicated its probable role in signaling [32]. Earlier findings have also indicated MG to play a regulatory role in many aspects of plants such as germination, root development, shoot morphogenesis, stomatal closure, photosynthesis, pollination and stress tolerance etc. [33,34,35,36]. At 300 mM NaCl a significant decrease in MG is observed in AtGLYI2 transgenic plants compared to AtGLYI3 and AtGLYI6. It has been shown earlier that even a minute decrease in MG can have significant effect on salinity stress tolerance [11].

Taken together, our results add further details to the literature of plant glyoxalases. We have shown that AtGLYI2, a Zn^2+^ dependent glyoxalase is the main enzyme involved in the detoxification of methylglyoxal in Arabidopsis. AtGLYI2’s extremely high enzymatic activity (5157 μmol/min/mg in comparison to AtGLYI3 (20.7 μmol/min/mg) and AtGLYI6 (7.68 μmol/min/mg) [12] provides it with an advantage to detoxify MG in AtGLYI2 overexpressing plants, despite the slight increase in its protein levels in comparison to AtGLYI3 and AtGLYI6 (Fig 1). Moreover, the T-DNA insertion mutants of AtGLYI2 seem to be affected a lot in its absence; consequently, showing very diminished growth and high sensitivity to presence of MG in the media. On the other hand, as AtGLYI3 and AtGLYI6 possess much lower enzymatic activities and their presence in the AtGLYI2 mutant plants is not being able to complement for the missing gene clearly demonstrates the major role of Zn^2+^ dependent GLYI in MG detoxification. Also, we report dose-dependent toxic effects of MG on the overall growth and development of WT, transgenic and mutant plants, as all plants irrespective of their genetic manipulation remain smaller in the media than the one supplemented without MG. Apart from this, we also show that overexpression of all three AtGLYI genes in Arabidopsis provides added advantage to withstand high saline conditions. Out of the three AtGLYIs, AtGLYI2 overexpressing transgenic plants maintain better growth rate, and were taller with more number of siliques under salinity stress conditions. This finding in Arabidopsis plants also align with the previous study conducted in *E*. *coli*, where cells overexpressing *AtGLYI2*, *AtGLYI3* and *AtGLYI6* genes were more stress tolerant as compared to control. Among the three, AtGLYI2 overexpressing cells were far more tolerant in presence of salinity (NaCl), oxidative (H_2_O_2_), exogenous MG, osmotic (mannitol) and heat stress [12]. Also, transgenic plants overexpressing AtGLYI2 maintained slightly lower levels of MG under control and salinity stress condition, emphasizing on its possible mechanism to resist the over accumulation of MG in the system. This possible resistance mechanism of over accumulating MG also correlates with the yeast complementation results, where yeast GLO1 mutant cells on being complemented with AtGLYI2 could withstand growth even at extremely high concentration of MG (4 mM) [12]. Finally, these finding lay emphasis on the role of Zn^2+^ dependent GLYIs in the plant system and indicate a positive relationship between them and salinity stress tolerance in plants.

Similar to rice OsGLYI-8, AtGLYI2 possesses a 28 amino acid nuclear localisation signal (NSL) at the N-terminal region and therefore should be involved in MG detoxification in the nucleus [13]. *In silico* subcellular localization of AtGLYI3 and AtGLYI6 revealed that they might be chloroplastic [28]. In future, an in-depth subcellular localization study of these three different GLYI might shed some light to their important roles. The finding of this study would be extremely helpful for anyone interested in engineering the Glyoxalase pathway in any crop plants in future. We have already shown that GLYI is the rate limiting enzyme in MG detoxification [17]. So, identifying the most important GLYI among three was crucial. Based on the previous studies, it is evident that majority of the plant species have both Zn^2+^ and Ni^2+^ dependent GLYI. The current study establishes potential of Zn^2+^ dependent GLYI for engineering Glyoxalase pathway for salinity stress tolerance in plant system. However, it would be important to explore the bioavailability of different metal ions in different parts of the plants, which might be critical to understand the role of multiple GLYI enzymes.

## Supporting information

S1 Raw Images Raw image of all the gels and blots used in the manuscript. (PDF)

S1 Fig Total leaf RNA and PCR amplification of *AtGLYI* genes. (PPTX)

S2 Fig Glyoxalse I domain position in AtGLYI2, AtGLYI3 and at AtGLYI6. (PPTX)

S1 Table List of primers and their respective sequences used for full-length amplification of *AtGLYI2*, *AtGLYI3* and *AtGLYI6* genes for gateway cloning. (DOCX)

S2 Table List of primers and their sequences used for confirmation of *AtGLYI* transgenic plants at genomic DNA level. (DOCX)

S3 Table List of primers used in PCR genotyping for identification of homozygous T-DNA insertion mutant. (DOCX)
