# Supplementary material for: Zn2+ dependent glyoxalase I plays the major role in methylglyoxal detoxification and salinity stress tolerance in plants
Source: PLoS One. 2020 May 26;15(5):e0233493. doi: 10.1371/journal.pone.0233493 (PMC7250436; doi:10.1371/journal.pone.0233493)
Supplement: S1 Raw Images — (PDF) [file pone.0233493.s001.pdf]

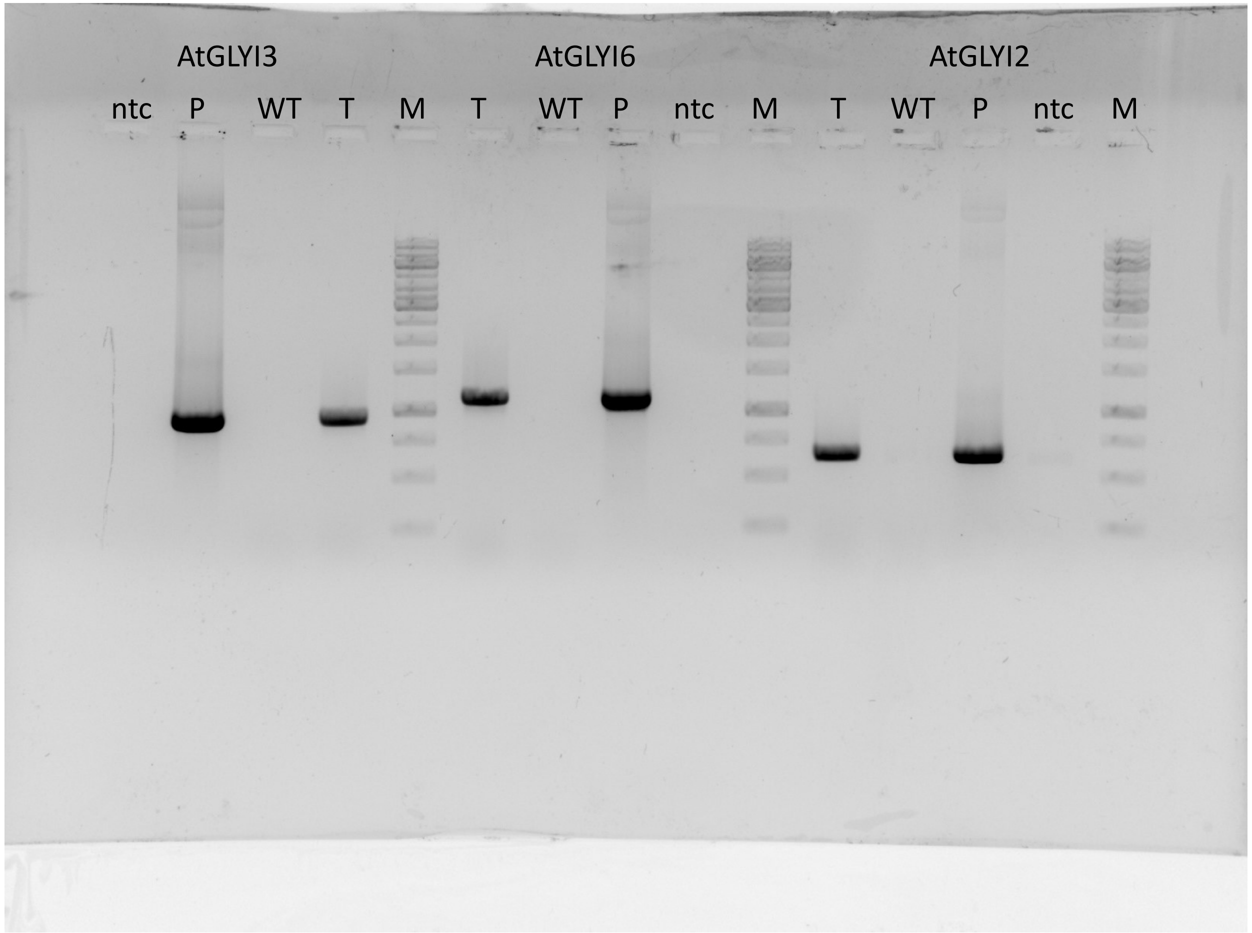

Raw gel image of **Figure 1** in the manuscript, confirming the integration of ATGLYI genes in Arabidopsis genomic DNA. Here WT: Wild type, P: Postive control; T: Transgenic; M: 1 Kb GeneRuler ladder.

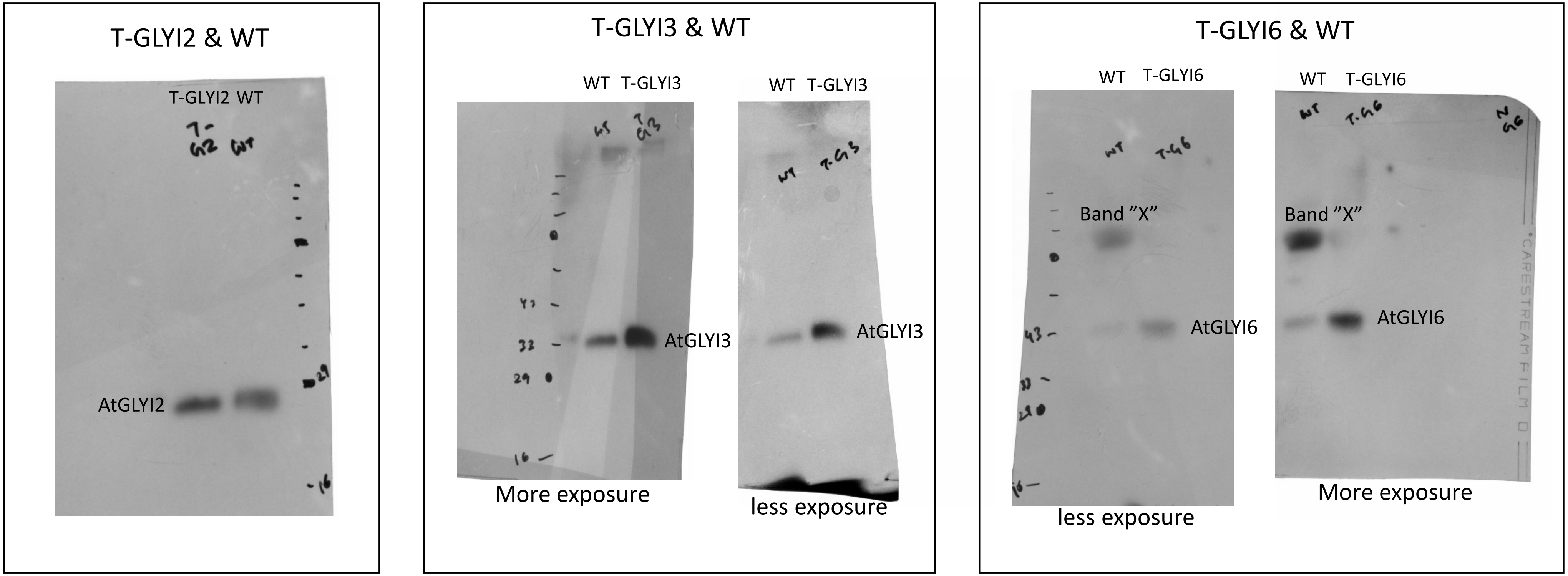

Raw western blot Images of **Figure 1** in the manuscript. Here WT: Wild type, T-GLYI2: AtGLYI2 transgenic, T-GLYI3: AtGLYI3 transgenic, T-GLYI6: AtGLYI6 transgenic, Band X: non-specific band of higher moleculer weight

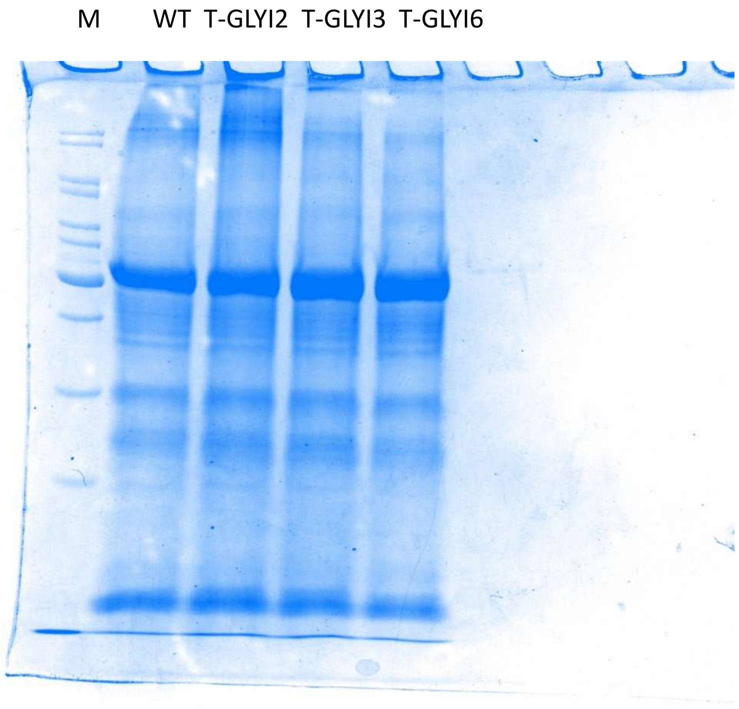

Raw image of SDS-PAGE gel of WT, AtGLYI2, AtGLYI3 and AtGLYI6 protein used in **Figure 1** in manuscript (loading control of western blot)

A

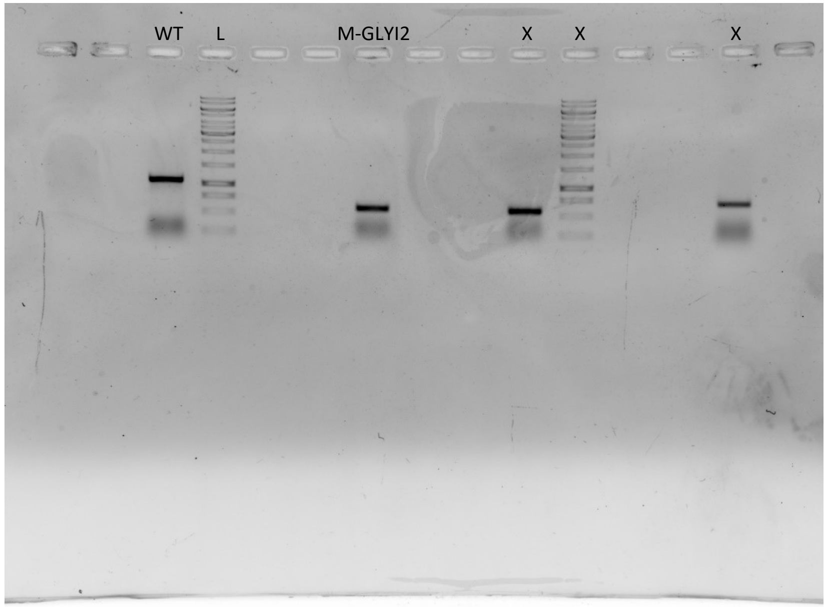

B

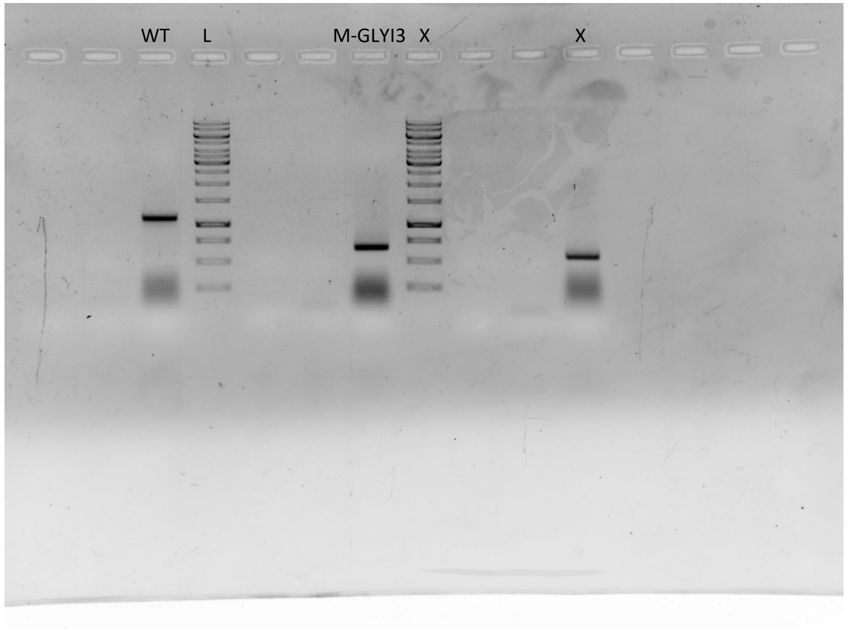

C

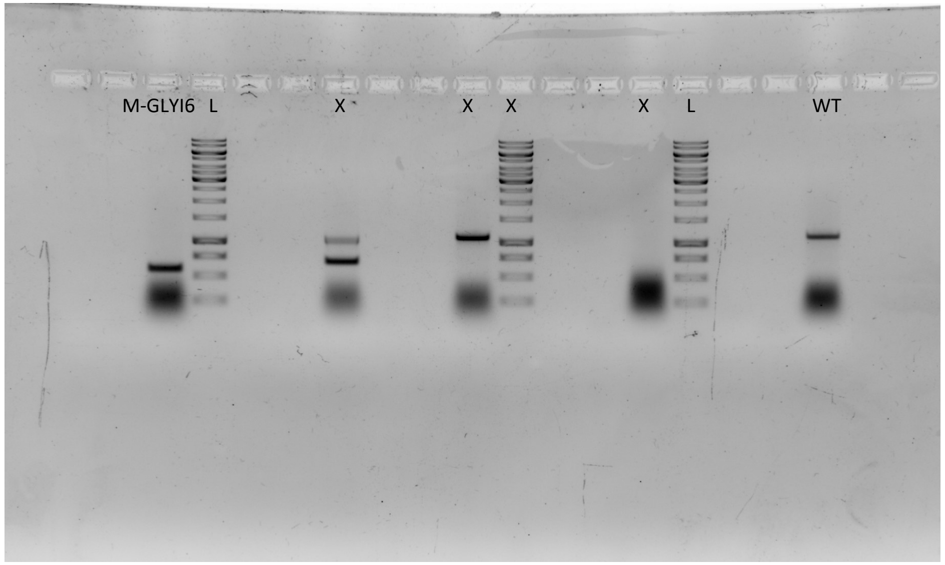

Raw gel Image for **Figure 2.** Here WT: Wild type; M-GLYI2, M-GLYI3 & M-GLYI6: Mutant line for AtGLYI2, AtGLYI3 & AtGLYI6, respectively. L: 1kb GeneRuler ladder.

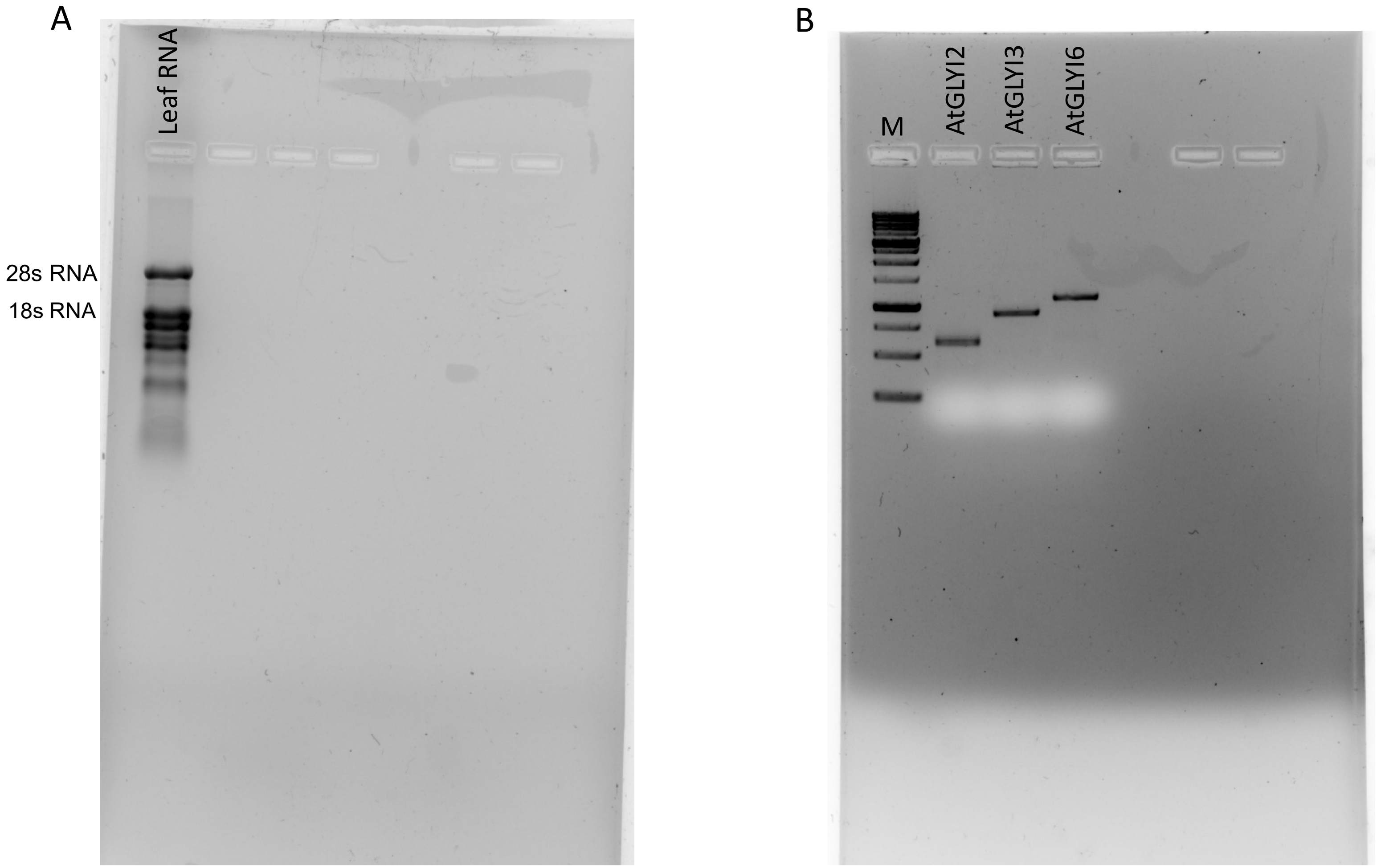

Raw gel image of Total leaf RNA (A) and PCR amplification of *AtGLYI* genes (B) used in Figure S2
