# Supplementary material for: Zn2+ dependent glyoxalase I plays the major role in methylglyoxal detoxification and salinity stress tolerance in plants
Source: PLoS One. 2020 May 26;15(5):e0233493. doi: 10.1371/journal.pone.0233493 (PMC7250436; doi:10.1371/journal.pone.0233493)
Supplement: S1 Fig — (PPTX) [file pone.0233493.s002.PPTX]

## Slide 1
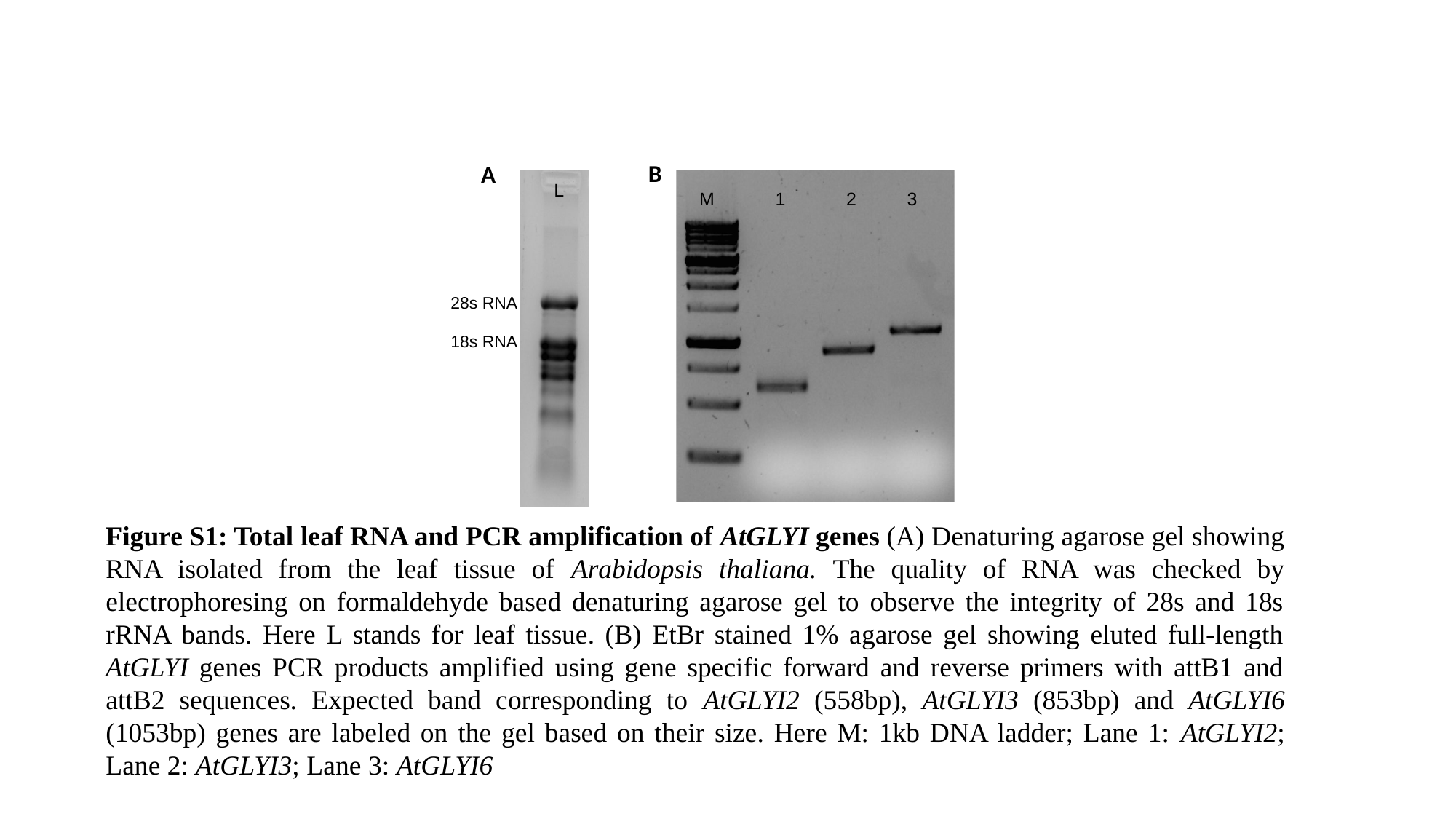

B
A
 M 1 2 3
28s RNA
18s RNA
L
Figure S1: Total leaf RNA and PCR amplification of AtGLYI genes (A) Denaturing agarose gel showing RNA isolated from the leaf tissue of Arabidopsis thaliana. The quality of RNA was checked by electrophoresing on formaldehyde based denaturing agarose gel to observe the integrity of 28s and 18s rRNA bands. Here L stands for leaf tissue. (B) EtBr stained 1% agarose gel showing eluted full-length AtGLYI genes PCR products amplified using gene specific forward and reverse primers with attB1 and attB2 sequences. Expected band corresponding to AtGLYI2 (558bp), AtGLYI3 (853bp) and AtGLYI6 (1053bp) genes are labeled on the gel based on their size. Here M: 1kb DNA ladder; Lane 1: AtGLYI2; Lane 2: AtGLYI3; Lane 3: AtGLYI6
