# Supplementary material for: Zn2+ dependent glyoxalase I plays the major role in methylglyoxal detoxification and salinity stress tolerance in plants
Source: PLoS One. 2020 May 26;15(5):e0233493. doi: 10.1371/journal.pone.0233493 (PMC7250436; doi:10.1371/journal.pone.0233493)
Supplement: S2 Fig — (PPTX) [file pone.0233493.s003.PPTX]

## Slide 1
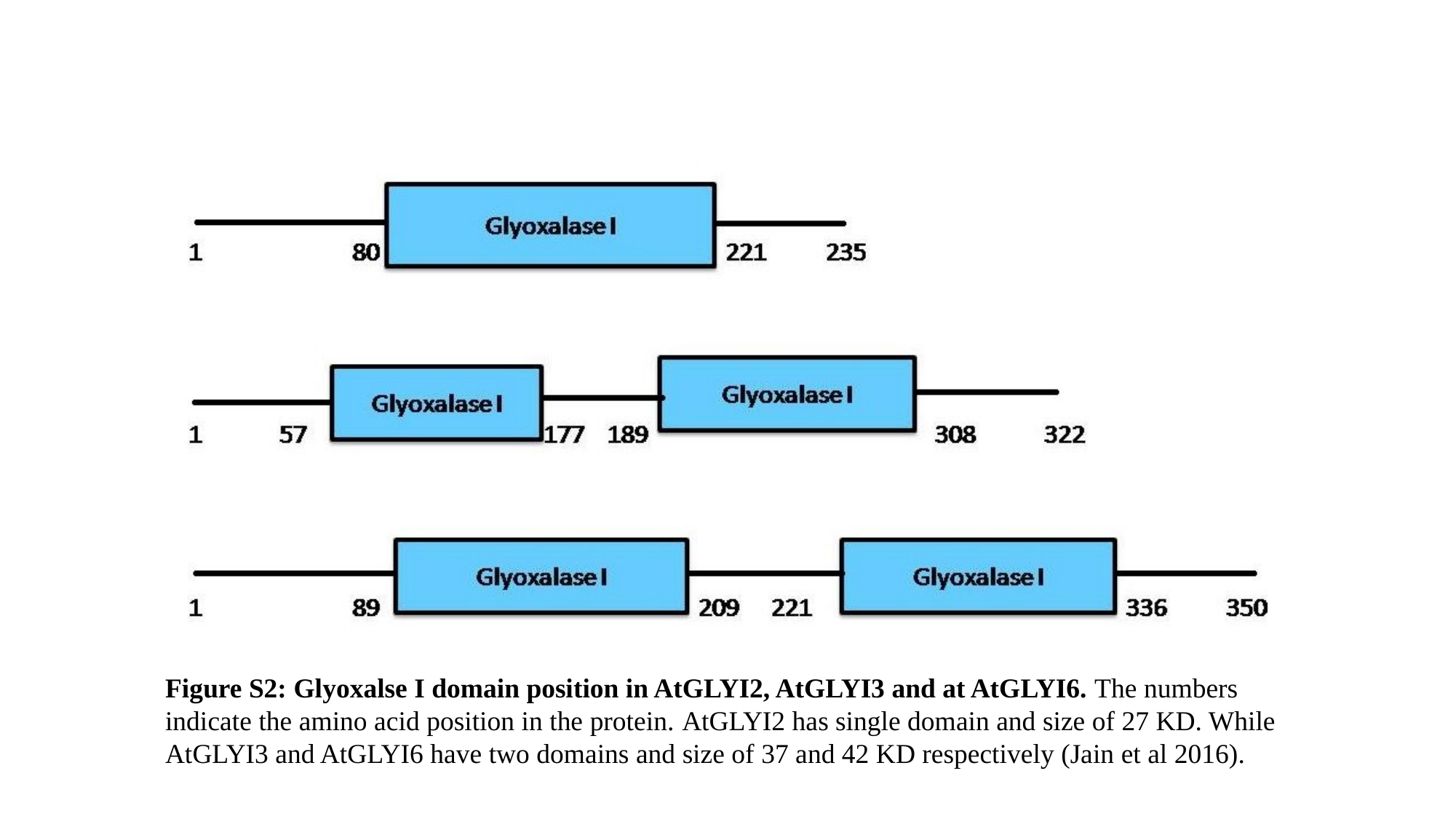

Figure S2: Glyoxalse I domain position in AtGLYI2, AtGLYI3 and at AtGLYI6. The numbers indicate the amino acid position in the protein. AtGLYI2 has single domain and size of 27 KD. While AtGLYI3 and AtGLYI6 have two domains and size of 37 and 42 KD respectively (Jain et al 2016).
