# Supplementary material for: Zn2+ dependent glyoxalase I plays the major role in methylglyoxal detoxification and salinity stress tolerance in plants
Source: PLoS One. 2020 May 26;15(5):e0233493. doi: 10.1371/journal.pone.0233493 (PMC7250436; doi:10.1371/journal.pone.0233493)
Supplement: S1 Table — (DOCX) [file pone.0233493.s004.docx]

**Supplementary Table S1: List of primers and their respective sequences used for full-length amplification of *AtGLYI2*, *AtGLYI3* and *AtGLYI6* genes for gateway cloning.** Here At: *Arabidopsis*, GI2/3/6: *AtGLYI2/3/6* respectively, F: Forward, R: Reverse, red color sequence: *attB1* and *attB2* sequences respectively, blue color sequence: extra nucleotides to be added for successful recombination, green color sequence: stop codon, B1: *attB1*, B2: *attB2*, SC: stop codon.

| Locus | Primer name | Primer sequence | Primer length |
| --- | --- | --- | --- |
| AT1G08110 | AtGI2-B1-F | GGGG ACAAGTTTGTACAAAAAAGCAGGCT ATGGCGTCGGAAGCGAGGGAATC | 52 bp |
|  | AtGI2-B2_SC-R | GGGG ACCACTTTGTACAAGAAAGCTGGGT C TCA AGCTGCGTTTACGGTAGTAGTT | 52 bp |
| AT1G11840 | AtGI3-B1-F | GGGG ACAAGTTTGTACAAAAAAGCAGGCT ATGGCT GAGGCTTCTGATTTGTT | 52 bp |
|  | AtGI3-B2_SC-R | GGGG ACCACTTTGTACAAGAAAGCTGGGT C TCA TTCCAGTTCCTTGAGAAAATCTTT | 54 bp |
| AT1G67280 | AtGI6-B1-F | GGGG ACAAGTTTGTACAAAAAAGCAGGCT ATGGTGAGGATCATTCCTATGGCT | 53 bp |
|  | AtGI6-B2_SC-R | GGGG ACCACTTTGTACAAGAAAGCTGGGT C TCA CTCCAGTTCTTTGAGAAAGTCA | 52 bp |
