# Supplementary material for: Zn2+ dependent glyoxalase I plays the major role in methylglyoxal detoxification and salinity stress tolerance in plants
Source: PLoS One. 2020 May 26;15(5):e0233493. doi: 10.1371/journal.pone.0233493 (PMC7250436; doi:10.1371/journal.pone.0233493)
Supplement: S2 Table — (DOCX) [file pone.0233493.s005.docx]

**Supplementary Table S2: List of primers and their sequences used for confirmation of *AtGLYI* transgenic plants at genomic DNA level.** Here At: *Arabidopsis*, GI2/3/6: *AtGLYI2/3/6* respectively, R: reverse, F: forward, green color sequence: XhoI restriction site, blue color sequence: extra nucleotide, 35S: 35S CaMV promoter, pE100: pEARLEY100 vector.

| Locus | Primer name | Primer sequence | Primer length |
| --- | --- | --- | --- |
| AT1G08110 | AtGI2 XhoI-R | CCGCTCGAG TCAAGCTGCGTTTACGGTAGTAGT T | 25 bp |
| AT1G11840 | AtGI3 XhoI-R | CCGCTCGAG TCATTCCAGTTCCTTGAGAAAATC TTT | 27 bp |
| AT1G67280 | AtGI6 XhoI-R | CCGCTCGAG TCACTCCAGTTCTTTGAGAAAGTC A | 25 bp |
|  | 35S_pE100_F | CGCAAGACCCTTCCTCTATATAAGG | 25 bp |
