# Supplementary material for: Zn2+ dependent glyoxalase I plays the major role in methylglyoxal detoxification and salinity stress tolerance in plants
Source: PLoS One. 2020 May 26;15(5):e0233493. doi: 10.1371/journal.pone.0233493 (PMC7250436; doi:10.1371/journal.pone.0233493)
Supplement: S3 Table — (DOCX) [file pone.0233493.s006.docx]

**Supplementary Table S3: List of primers used in PCR genotyping for identification of homozygous T-DNA insertion mutant.** Here Mut: Mutant, G2: *AtGLYI2*, G3: *AtGLYI3*, G6: *AtGLYI6*, pROK2, pCSA110, pDAP101, pDs-Lox: name of vectors used for insertion of T-DNA into the plant genome, LB: left border, RP: right primer, LP: left primer.

| S.No. | Primer name | Primer sequence | Primer length | Tm | GC% |
| --- | --- | --- | --- | --- | --- |
| AtGLYI2 mutant lines | | | | | |
| 1. | pROK2_LBb1.3 _new | ATTTTGCCGATTTCGGAAC | 19 bp | 47 | 42 |
| 2. | Mut_G2-1,2_RP | ATTCGGAAAAACCGTCTTTTG | 21 bp | 60.33 | 38.1 |
| 3. | Mut_G2-1,2_LP | TGCCCTGTGACAACAACATAG | 21 bp | 59.62 | 47.62 |
| 4. | Mut_G2-3_pCSA110_LB3 | TAGCATCTGAATTTCATAACCAA  TCTCGATACAC | 34 bp | 60 | 35 |
| 5. | Mut_G2-3_RP | CGTGGAAAGCGAAAACTACAG | 21 bp | 59.93 | 47.62 |
| 6. | Mut_G2-3_LP | GGGAGCTTTAAATATGGTGGC | 21 bp | 59.82 | 47.62 |
| 7. | Mut_G2-4_pDAP101_LB3 | TAGCATCTGAATTTCATAACCA  ATCTCGATACAC | 34 bp | 60 | 35 |
| 8. | Mut_G2-4_RP | TGGCGAACCATAAACAAAATC | 21 bp | 59.82 | 38.1 |
| 9. | Mut_G2-4_LP | TCCTTGGATTAGCAATTCGTG | 21 bp | 60.08 | 42.86 |
| AtGLYI3 mutant lines | | | | | |
| 1. | Mut_G3-1_pDs-Lox_LB | AACGTCCGCAATGTGTTATTA  AGTTGTC | 28 bp | 57 | 39 |
| 2. | Mut_G3-1_RP | TGGGGTATGCTGAAGAATACG | 21 bp | 59.97 | 47.62 |
| 3. | Mut_G3-1_LP | TTGTGGCTGAGTTTTCATGTG | 21 bp | 59.75 | 42.86 |
| 4. | Mut_G3-2_RP | TATCGATCCACGAATCCTGTC | 21 bp | 59.91 | 47.62 |
| 5. | Mut_G3-2_LP | GCCAATCTCCTGAAACAATTTAG | 23 bp | 59.18 | 39.13 |
| 6. | Mut_G3-3_RP | TTCTAAATGGGCTGAGATCCC | 21 bp | 60.4 | 47.62 |
| 7. | Mut_G3-3_LP | CAACAAATCAGAAGCCTCAGC | 21 bp | 60.01 | 47.62 |
| AtGLYI6 mutant lines | | | | | |
| 1. | Mut_G6-1_RP | CGATGTCGTTTGACATGTTTC | 21 bp | 59.04 | 42.86 |
| 2. | Mut_G6-1_LP | ATTTGCAATGCCTCACAAATC | 21 bp | 59.96 | 38.1 |
| 3. | Mut_G6-2_RP | GATGATTACGGAGAGTGCGAG | 21 bp | 59.85 | 52.38 |
| 4. | Mut_G6-2_LP | GCATCGAGAAATTCTCACCAC | 21 bp | 59.69 | 47.62 |
| 5. | Mut_G6-3_RP | TCGAGATTCAGAGCTCTCTGC | 21 bp | 59.99 | 52.38 |
| 6. | Mut_G6-3_LP | AGCAACACCAACAACGGATAC | 21 bp | 59.91 | 47.62 |
| 7. | Mut_G6-4_RP | TACAGTTTTCGCCACCTTTTG | 21 bp | 60.15 | 42.86 |
| 8. | Mut_G6-4_LP | GTCGTCACTTAATCTCCGACG | 21 bp | 59.75 | 52.38 |
| 9. | Mut_G6-5_RP | AGGAGAATCAGAGAAGCAGGC | 21 bp | 60.11 | 52.38 |
| 10. | Mut_G6-5_LP | AGCAACACCAACAACGGATAC | 21 bp | 59.91 | 47.62 |
| 11. | Mut_G6-6_RP | CACAGGAAAGCCAAAGTGAAG | 21 bp | 59.9 | 47.62 |
| 12. | Mut_G6-6_LP | TCTACAATGGTGTCCCTAGCG | 21 bp | 60.14 | 52.38 |
